# From Neonatal Cholestasis to Progressive Neurological Impairment: A Case of Niemann-Pick Disease Type C

**DOI:** 10.7759/cureus.110744

**Published:** 2026-06-12

**Authors:** Liliana Carvalho de Sousa, Cecília Gomes Pereira, Catarina Magalhães

**Affiliations:** 1 Department of Pediatrics, Unidade Local de Saúde do Alto Ave, Guimarães, PRT; 2 Department of Pediatrics, Unidade Local de Saúde de Barcelos/Esposende, Barcelos, PRT

**Keywords:** hepatosplenomegaly, lysosomal storage disorder, miglustat, neonatal cholestasis, neurodevelopmental delay, niemann-pick disease type c, oxysterols, pediatric neurodegeneration

## Abstract

Physiological neonatal jaundice typically resolves within the first two weeks of life. Persistence beyond this period should prompt evaluation for neonatal cholestasis, which may represent the first manifestation of an underlying metabolic disorder.

We report a six-year-old boy who presented at two months of age with cholestatic jaundice, hepatosplenomegaly, and elevated transaminases. Initial findings suggested congenital cytomegalovirus infection; however, liver biopsy was nonspecific, and further investigations were negative, leading to a diagnosis of idiopathic giant cell hepatitis. Although jaundice resolved, hepatosplenomegaly persisted. Developmental delay became apparent at three years, followed by progressive neurological impairment, including ataxia, dysphagia, and vertical supranuclear gaze palsy. Biochemical testing revealed elevated oxysterols, and molecular analysis confirmed Niemann-Pick disease type C (NPC) due to pathogenic *NPC1* variants. Treatment with miglustat resulted in transient clinical improvement, and the patient was subsequently enrolled in a phase III clinical trial evaluating N-acetyl-L-leucine.

Niemann-Pick disease type C should be considered in children with unexplained neonatal cholestasis and progressive neurological deterioration, as early recognition may enable timely diagnosis and access to disease-modifying therapies, potentially improving clinical outcomes.

## Introduction

Niemann-Pick disease comprises a heterogeneous group of autosomal recessive lysosomal storage disorders historically classified into types A, B, and C. Types A and B, currently referred to as acid sphingomyelinase deficiency, result from pathogenic variants in the *SMPD1* gene leading to acid sphingomyelinase deficiency [1]. In contrast, Niemann-Pick disease type C (NPC) is caused by pathogenic variants in the *NPC1* or *NPC2* genes, resulting in impaired intracellular lipid trafficking and progressive accumulation of cholesterol and glycosphingolipids in multiple tissues [2,3]. The clinical presentation is highly heterogeneous, ranging from severe neonatal disease to chronic neurodegeneration with onset in childhood or adulthood [2]. Early manifestations often include neonatal cholestasis and hepatosplenomegaly, which may precede neurological symptoms by several years and frequently lead to diagnostic delay [2].

Early diagnosis remains challenging due to the heterogeneous and often nonspecific clinical presentation, while currently available therapeutic options remain limited, particularly for neurological manifestations.

We report a case of late infantile form of Niemann-Pick disease type C (NPC) presenting with neonatal cholestasis followed by progressive neurological impairment, highlighting the diagnostic challenges and the importance of early clinical suspicion.

## Case presentation

A six-year-old boy, born to non-consanguineous parents, was referred for evaluation of global developmental delay and progressive neurological impairment. Family history was notable for a cousin with an undiagnosed neurodevelopmental delay who died following an epileptic seizure in a swimming pool.

The patient presented at two months of age with cholestatic jaundice associated with hepatosplenomegaly and elevated transaminases. Initial evaluation raised suspicion for cytomegalovirus infection (positive IgM, negative IgG), and liver biopsy revealed nonspecific findings with mildly positive polymerase chain reaction (PCR) for cytomegalovirus. Extensive workup, including screening for celiac disease, Wilson disease, and alpha-1 antitrypsin deficiency, was negative. A diagnosis of idiopathic giant cell hepatitis was assumed.

During early childhood, no further episodes of jaundice were reported, although hepatosplenomegaly and elevated transaminases persisted but gradually improved.

The patient achieved independent walking at 15 months and spoke his first words at 18 months. Developmental delay became evident from the age of three years, characterized by absence of symbolic play, inability to use toys according to their intended purpose, and limited vocabulary. At six years of age, he demonstrated developmental regression, including worsening play skills and language deterioration. Behavioral abnormalities included emotional dysregulation, short attention span, impulsivity, and episodes of coprolalia. Progressive loss of autonomy in activities of daily living was also noted, including toileting.

Neurological symptoms progressively worsened from six years of age, with frequent falls, impaired visual tracking, and compensatory head movements when attempting downward gaze. The patient was described as clumsy and experienced episodes of choking during feeding. Neurological examination revealed language and behavior inappropriate for age. Cranial nerve examination demonstrated limited saccadic eye movements, impaired horizontal gaze, and vertical supranuclear gaze palsy. Cerebellar examination revealed ataxia.

Given the combination of neonatal cholestasis, hepatosplenomegaly, and progressive neurological deterioration with characteristic ocular findings, NPC was suspected. Biochemical analysis demonstrated elevated oxysterols, including 7-ketocholesterol and cholestane-3β,5α,6β-triol, as well as increased lysosphingomyelin and lyso-SM-509, supporting the diagnosis. These findings are summarized in Table 1.

**Table 1:** Biochemical findings supporting the diagnosis of Niemann-Pick disease type C MoM: multiples of the median, Lyso-SM-509: lysosphingomyelin-509

Biomarker	Result	Reference value
7-ketocholesterol	647 ng/mL	0-301 ng/mL
Cholestane-3β,5α,6β-triol	117 ng/mL	0-42.6 ng/mL
Lysosphingomyelin	8.9 nmol/L	<7.0 nmol/L
Lyso-SM-509	92 MoM	<2.7 MoM

**Figure 1:**
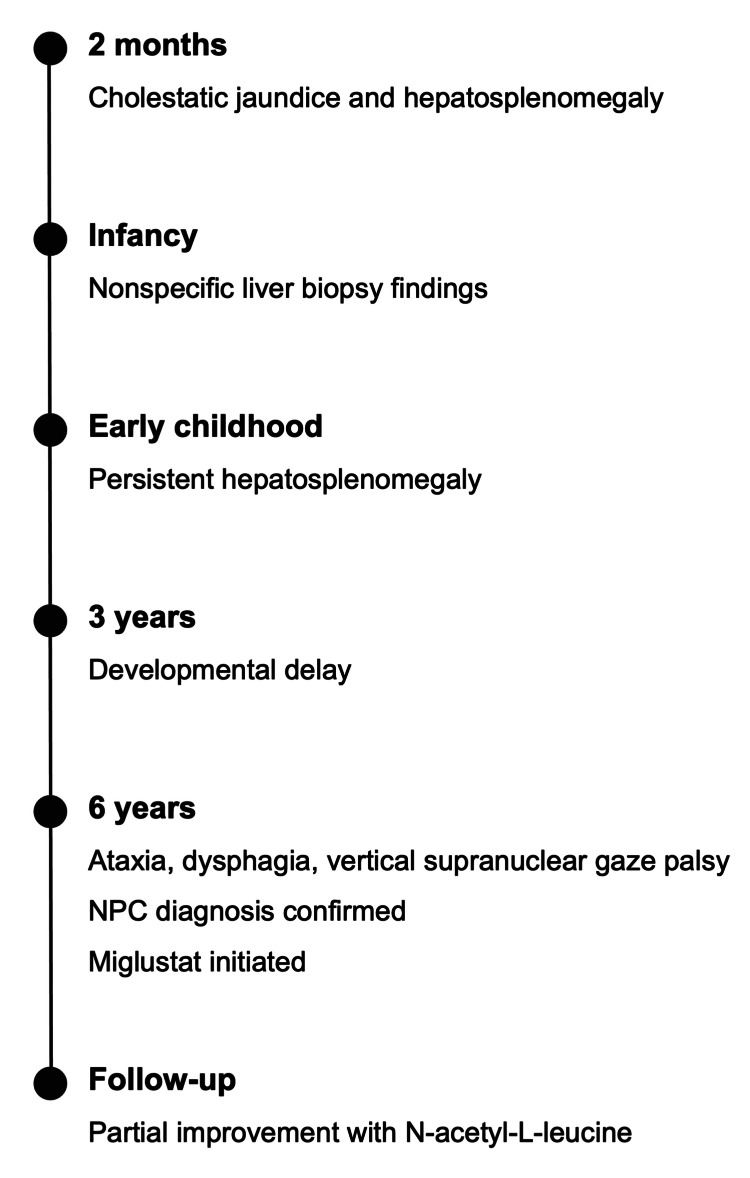
Clinical timeline and disease progression NPC: Niemann-Pick disease type C

Brain magnetic resonance imaging (MRI) demonstrated bilateral white matter abnormalities with T2/fluid-attenuated inversion recovery (FLAIR) hyperintensity predominantly involving the parietal white matter and posterior corona radiata, suggestive of hypomyelination. Mild cortical atrophy and a globally thin but complete corpus callosum were also observed. Molecular genetic testing identified two previously reported heterozygous pathogenic variants in the *NPC1* gene, c.3019C>G (p.P1007A) in exon 20 and c.3104C>T (p.A1035V) in exon 21, confirming the diagnosis of NPC. The patient’s clinical course and disease progression are illustrated in Figure 1.

Treatment with miglustat was initiated at six years of age, with initial improvement in gait, ocular function, and swallowing. However, progressive cognitive and behavioral decline was observed over time, with a fluctuating neurological course.

The patient was later enrolled in a phase III clinical trial evaluating N-acetyl-L-leucine, with partial improvement in communication, behavior, and swallowing during follow-up, demonstrating improved responsiveness, better balance, and absence of dysphagia, although cataplexy emerged. At the most recent evaluation, the patient demonstrated improved gait under supervision, persistent oral exploratory behavior, severely limited language with rare verbalization, and an inability to follow simple commands.

## Discussion

NPC is a rare autosomal recessive lysosomal storage disorder caused by pathogenic variants in the *NPC1* and *NPC2* genes, accounting for approximately 95% and 5% of cases, respectively [2]. The disease results from impaired intracellular trafficking and processing of low-density lipoprotein-derived cholesterol and other lipids, including glycosphingolipids and sphingosine, leading to their accumulation in multiple tissues, particularly within the central nervous system, and progressive neurodegeneration [2].

NPC is estimated to affect at least 1 in 120,000 live births in Europe [2,4], although it is likely underdiagnosed due to its heterogeneous and often nonspecific clinical presentation [5]. The disease may manifest at any age, ranging from a rapidly progressive and often fatal neonatal form to a chronic neurodegenerative disease with onset in childhood or adulthood [2].

Neonatal cholestasis and hepatosplenomegaly are among the most common early manifestations of NPC and may precede neurological symptoms by several years. In many cases, jaundice resolves spontaneously, while organomegaly persists, contributing to a misleading impression of clinical resolution [2,6]. In the present case, the initial diagnosis of idiopathic giant cell hepatitis illustrates the diagnostic challenge of NPC in early life, particularly when findings are nonspecific or partially explained by alternative conditions. This case underscores the importance of considering NPC in infants presenting with unexplained cholestasis, even when initial findings suggest alternative diagnoses.

The subsequent development of neurological symptoms, including developmental delay, ataxia, dysphagia, behavioral disturbances, and vertical supranuclear gaze palsy, is highly suggestive of the infantile form of NPC [4,7]. Among these, vertical supranuclear gaze palsy, observed in this patient, is one of the most characteristic early neurological signs and should prompt consideration of this diagnosis [4,7]. Additional features, such as cataplexy, although not always present, further support clinical suspicion and are relatively uncommon in other neurodegenerative disorders. Hearing impairment and seizures may also occur during disease progression, and epilepsy is associated with a worse prognosis [7].

In pediatric patients, NPC is commonly classified according to the age of onset of neurological symptoms into early infantile, late infantile, and juvenile forms. The late infantile form, typically presenting between two and six years, is characterized by early visceral involvement followed by progressive neurological deterioration [4,5,7]. The clinical course observed in our patient is consistent with this phenotype.

The combination of developmental delay and a history of neonatal cholestasis or hepatosplenomegaly should raise a strong clinical suspicion for NPC [8]. Diagnostic confirmation relies on a combination of biochemical and molecular genetic testing [3,4]. Biomarkers such as oxysterols and lysosphingomyelin derivatives, including lyso-SM and lyso-SM-509 (also known as N-palmitoyl-O-phosphocholineserine, PPCS), are sensitive and specific tools for initial screening [3,4,9,10]. Molecular genetic analysis remains the gold standard for definitive diagnosis [3,4].

Histopathological findings may support the diagnosis but are not specific, and conventional light microscopy may fail to detect NPC in cases presenting as neonatal cholestasis. In selected cases, filipin staining of cultured fibroblasts can be used, although this technique is time-consuming and has largely been replaced by biochemical and molecular methods [11].

Neuroimaging findings in NPC are variable and often nonspecific. Brain MRI in our patient demonstrated white matter abnormalities suggestive of hypomyelination, mild cortical atrophy, and a thin corpus callosum. Although such findings are not diagnostic, white matter changes and cerebral atrophy have been reported in patients with NPC and may support the diagnosis in the appropriate clinical context [7].

NPC remains an incurable disease associated with progressive neurological decline and premature mortality [7]. Management is primarily supportive and requires a multidisciplinary approach involving pediatric neurology, gastroenterology/hepatology, nutritional support, physical and speech therapy, and psychological care [4]. Premature mortality is mainly related to progressive neurological deterioration and its complications, particularly dysphagia, aspiration pneumonia, and respiratory infections [4,7]. Compared with the late infantile form observed in this patient, the early infantile phenotype is characterized by earlier neurological onset, more rapid disease progression, and a poorer prognosis [4,7]. Therefore, early recognition is essential to enable timely diagnosis, appropriate multidisciplinary management, and access to emerging therapeutic strategies. This case highlights the importance of recognizing early clinical red flags, particularly neonatal cholestasis followed by progressive neurological symptoms, to avoid diagnostic delay and improve patient care.

## Conclusions

This case highlights the need for increased clinical awareness of NPC, particularly in children with a history of neonatal cholestasis and progressive neurological symptoms. Early recognition is essential to enable timely diagnosis, initiation of therapy, and appropriate genetic counseling, with the potential to improve clinical outcomes.

NPC should be considered in the differential diagnosis of children presenting with unexplained cholestasis followed by neurological deterioration.
